# Supplementary material for: National Experiences from 30 Years of Provider-Mediated Cascade Testing in Lynch Syndrome Families—The Danish Model
Source: Cancers (Basel). 2024 Apr 20;16(8):1577. doi: 10.3390/cancers16081577 (PMC11048852; doi:10.3390/cancers16081577)
Supplement: Supplementary file 1 [file cancers-16-01577-s001.zip › Supplementary Figure S1.pdf]

# WRITTEN CONSENT

# FOR SHARING DATA IN RELATION TO MY DISEASE

About me ☐ Regarding my child ☐ Regarding a person lacking legal capacity ☐

Deceased family member ☐ Family relation ☐ Year at death:_________

Social security number:________________________ Tell:_______________________________

Name:__________________________ E-mail:_________________________________

Treatment at following hospital:____________________________________________________________

Diagnoses, year:_________________________________________________________________________

Information from medical journals, x-ray images, laboratory analyses etc. can be forwarded to:

Clinical Genetic Department [Hospital] Yes ☐ No ☐

- in case the consent form is collected in relation to cancer surgery

Clinical Genetic Departments in relation to counselling of family relatives Yes ☐ No ☐

Tissue from the surgery may be analyzed for relevant biomarkers Yes ☐ No ☐

- in case the consent form is collected in relation to cancer surgery

Material from blood samples, abovementioned tissue etc. can be stored for future analyses Yes ☐ No ☐

Information can be forwarded to:

Breast cancer database Yes ☐ No ☐

HNPCC Register (for hereditary colorectal cancer) Yes ☐ No ☐

(Detailed information on the registers will be given at the genetic counselling at the Clinical Genetic Department)

Date:___________________ Signature:_______________________________________

Medical doctor:_____________________________
