# Supplementary figures and images for: National Experiences from 30 Years of Provider-Mediated Cascade Testing in Lynch Syndrome Families—The Danish Model
Source: Cancers (Basel). 2024 Apr 20;16(8):1577. doi: 10.3390/cancers16081577 (PMC11048852; doi:10.3390/cancers16081577)

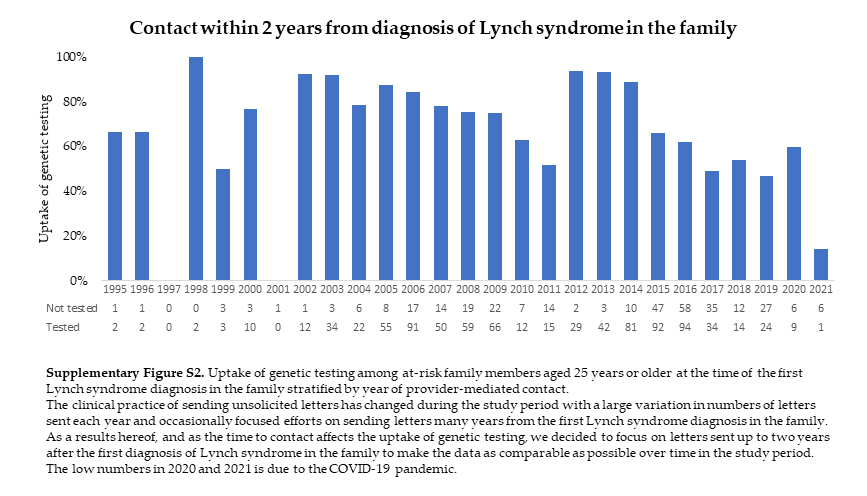

Supplement: Supplementary file 1 [file cancers-16-01577-s001.zip › Supplementary Figure S2.tif]
